# Tertiary Survey bei Traumapatienten: Praktische Empfehlungen der Sektion Trauma der DIVI

**DOI:** 10.1007/s00113-025-01600-y

**Published:** 2025-09-01

**Authors:** Paula Beck, Aileen Spieckermann, Jörg Bayer, Oliver Cruciger, Hanns-Christoph Held, Katharina Hinrichs, Axel Rand, Uwe Hamsen, Sektion Trauma der DIVI

**Affiliations:** 1 Klinik für Unfallchirurgie und Orthopädie, Schwarzwald-Baar Klinikum Villingen-Schwenningen GmbH, Klinikstr. 11, 78052 Villingen-Schwenningen, Deutschland; 2 Chirurgische Klinik, BG Universitätsklinikum Bergmannsheil, Bochum, Deutschland; 3 Klinik für Allgemein- Visceral- und Gefäßchirurgie, Universitätsklinikum Dresden, Dresden, Deutschland; 4 Zentrum für Notfallmedizin, Städtisches Klinikum Dresden, Dresden, Deutschland; 5 Klinik für Anästhesiologie und Intensivmedizin, Universitätsklinikum Dresden, Dresden, Deutschland; 6 Chirurgische Klinik, BG Universitätsklinikum Bergmannsheil, Bochum, Deutschland

**Keywords:** Polytrauma, Schwerverletztenversorgung, Übersehene Verletzungen, Verspätet diagnostizierte Verletzungen, 3. Untersuchungsgang, Polytrauma, Trauma Care, Missed injuries, Delayed diagnosed injuries, Tertiary examination

## Abstract

Der Tertiary Survey dient der vollständigen Erfassung von Verletzungsfolgen in den Stunden und Tagen nach dem Eintritt eines Polytraumas. Ein strukturierter Prozess führt erwiesenermaßen zu einer hohen Rate bis dahin übersehener zusätzlicher Diagnosen und zu daraus folgenden Änderungen des weiteren Prozedere. Dennoch gibt es für den Tertiary Survey bisher keine konkreten praktischen Empfehlungen oder Durchdringung in deutschen Traumazentren. Wir empfehlen eine sehr frühzeitige erstmalige Durchführung des Tertiary Survey auf der Intensivstation, gefolgt von ggf. häufigen Wiederholungen, wenn sich der klinische Zustand des Patienten/der Patientin verändert. Wir empfehlen, den Tertiary Survey zusätzlich als strukturierte Reevaluation des Patienten/der Patientin über die körperliche Untersuchung hinaus zu nutzen. Dabei sollen psychische Belastung evaluiert, bildgebende und konsiliarische Befunde auf Vollständigkeit überprüft sowie zusätzliche Informationen zu Unfall und Fremdanamnese eingeholt werden. Nach der Zusammentragung aller Informationen soll das weitere Prozedere evaluiert bzw. festgelegt werden. Die Sektion Trauma der DIVI entwickelt einen frei verfügbaren Bogen für den Tertiary Survey, der einer kontinuierlichen Optimierung unter wissenschaftlicher Begleitung unterliegt. Langfristig soll dadurch ein Instrument zur standardisierten Implementierung in die lokalen Verfahrensanweisungen der jeweiligen Traumazentren, das klinikinterne und übergeordnete Qualitätsmanagement sowie in die digitale Notfallkette geschaffen werden.

**Zusatzmaterial online:**

In der Online-Version dieses Beitrags (10.1007/s00113-025-01600-y), erreichbar über den QR-Code unten, finden Sie den von der Sektion Trauma der DIVI entwickelten Untersuchungsbogen für den Tertiary Survey zum Ausdrucken (mit freundlicher Genehmigung der DIVI).

## Abstract (en)

The tertiary survey serves to identify injuries and their consequences in the hours and days following polytrauma. A structured process has been proven to result in a high rate of previously missed additional diagnoses, leading to subsequent changes in patient management; however, to date there are no concrete practical recommendations or widespread implementation of the tertiary survey in German trauma centers. We recommend performing the tertiary survey for the first time as early as possible in the intensive care unit, followed by repeated assessments as needed when the patient´s clinical condition changes. Furthermore, we recommend utilizing the tertiary survey as a structured re-evaluation of the patient beyond physical examination. This should include the assessment of psychological stress, a thorough review of imaging and consultative findings and the collection of additional information regarding the accident and the patient´s medical history. After compiling all information further treatment plans should be evaluated and defined. The Trauma Section of the German Interdisciplinary Association for Intensive and Emergency Medicine (DIVI) is developing a freely available tertiary survey form, which will undergo continuous optimization supported by scientific evaluation. In the long term, the goal is to provide a standardized tool for integration into local standard operating procedures of trauma centers, quality management systems and digital emergency care pathways.

## Hintergrund

Die Versorgung schwer verletzter Patient:innen erfolgt gemäß der S3-Leitlinie Schwerverletztenversorgung [2]. Hierin fest verankert ist der sognannte Primary Survey (oder 1. Untersuchungsgang), welcher prioritätenorientiert nach dem ABCDE-Schema erfolgt und der dazu dienen soll, unmittelbar lebensbedrohliche Zustände zu erkennen und zu behandeln. An die initiale Schockraumversorgung schließt sich der sog. Secondary Survey (oder 2. Untersuchungsgang) an, sofern nicht die Indikation für einen unmittelbar notwendig werdenden operativen Eingriff bzw. eine unmittelbar notwendig werdende Intervention gestellt wird. Im Secondary Survey sollen die Patient:innen von Kopf bis Fuß untersucht werden, und unter Würdigung der erfolgten apparativen Diagnostik sollen am Ende des Secondary Survey alle erlittenen Verletzungen dokumentiert sein. Je nach Verletzungsmuster werden die Patient:innen dann im OP, auf einer Intensiv- bzw. Überwachungsstation oder auf der Normalstation weiterbehandelt.

Der Fokus der bis zur Aufnahme auf die (Intensiv‑)Station stattfindenden Diagnostik und Therapie liegt auf dem Prinzip „treat first what kills first“ [2]. Auch bei akut notwendig werdender Reevaluation von Traumapatient:innen im Verlauf des stationären Aufenthalts, insbesondere bei einer plötzlichen Zustandsverschlechterung, empfiehlt sich das Vorgehen nach dem ABCDE-Schema.

Unabhängig hiervon und parallel zu anderen intensivmedizinischen Maßnahmen sollte jedoch eine ausführliche, nicht unter Zeitdruck stehende, regelhafte Reevaluation der Patient:innen mit einer ausführlichen körperlichen Untersuchung erfolgen, der Tertiary Survey (Syn. 3. Untersuchungsgang, 3rd Survey).

In einer aktuellen Studie aus dem TraumaRegister DGU® konnten Gümbel et al. zeigen, dass bei 9,2 % der Patienten relevante Verletzungsfolgen erst auf der Intensivstation festgestellt wurden, davon 36 % im Bereich des Kopfes, 33 % im Bereich der Extremitäten und 20 % am Thorax [6].

Eine strukturierte Untersuchung innerhalb der ersten 72 h nach dem Traumaeintritt kann die Zahl der verzögert festgestellten Diagnosen senken („delayed diagnosed injuries“, [4, 11]). Des Weiteren können im Rahmen eines strukturierten Tertiary Survey Befunde, welche zu einer relevanten Änderung des Prozedere führen, erhoben werden [8].

Der 3. Untersuchungsgang gilt also 3 verschiedenen Aspekten:

1. Es sollen bisher nichterkannte Verletzungen detektiert werden („delayed detected diagnosis“).
2. Es sollen dynamische Befunde sowie graduelle Zustandsveränderungen erkannt werden. Beispiele sind ein größenprogredientes Hämatom oder ein zunehmender Sensibilitätsverlust.
3. Es sollen Traumafolgen erkannt werden, die erst im Verlauf entstanden sind, z. B. ein Kompartmentsyndrom oder ein Gefäßverschluss.

Klare evidenzbasierte Empfehlungen zum exakten Zeitpunkt, der genauen Abfolge, der Qualifikation der Durchführenden oder gar Für und Wider einzelner verschiedener Untersuchungsprotokollbogen gibt es jedoch bisher nicht. Die S3-Leitlinie Intensivtherapie nach Polytrauma gibt zu diesem Themenkomplex folgende Empfehlung: „Um die Relevanz dieses Prozesses hervorzuheben, wurde eine Empfehlung im Expert:innenkonsens formuliert: Konsensbasierte Empfehlung (GPP): Protokolle zur optimierten Durchführung und Dokumentation von Tertiäruntersuchung können genutzt werden (starker Konsens)“ [3].

Die Sektion Trauma der DIVI hat im Sinne einer Expertenempfehlung Vorschläge für das praktische Vorgehen beim Tertiary Survey erarbeitet. Hierbei sollten all die Patient:innen, bei denen im Rahmen der stationären Aufnahme nach ATLS-Algorithmus ein Primary Survey und ein Secondary Survey durchgeführt wurde, einen Tertiary Survey erhalten.

## Praktisches Vorgehen

### Zeitpunkt der Untersuchung

Der Zeitpunkt des 3. Untersuchungsganges sollte grundsätzlich im ausreichenden Abstand zum Zeitpunkt des Unfalls und auch zum Zeitpunkt des zweiten Untersuchungsganges im Schockraum geschehen. Das heißt, sollte der Patient/die Patientin nach der Erstversorgung im Schockraum mehrere Stunden im OP gewesen sein, kann der 3. Untersuchungsgang zügig innerhalb von 1–2 h nach der Aufnahme auf der Intensivstation erfolgen. Sollte der Patient/die Patientin innerhalb von 1–3 h nach dem Unfall bereits auf der Intensiv- oder Normalstation aufgenommen sein, sollte der 3. Untersuchungsgang 6–12 h nach dem Unfall erfolgen.

*Eine ausführliche Untersuchung unter Einbeziehung aller Aspekte des 3. Untersuchungsganges sollte erstmalig innerhalb von 6–12* *h nach Unfall erfolgen.*

Das bedeutet nicht, dass fokussierte Untersuchungen nicht auch deutlich eher erfolgen können oder müssen. Beispiele sind engmaschige neurologische Untersuchungen, Überwachungen bei kritischer Perfusion von Körperteilen oder die engmaschige fokussierte Abdomenuntersuchung bei nichtoperativem Management von Bauchorganlazerationen.

*Der 3. Untersuchungsgang muss (ggf. mehrmals) innerhalb der nächsten Tage auf der Intensivstation wiederholt werden.*

In vielen Situationen kann selbst ein ausführlich und gewissenhaft durchgeführter 3. Untersuchungsgang nicht ausreichend sein: Sedierte, narkotisierte oder delirante Patient:innen können keine Angaben zu Schmerzen oder Beschwerden wie etwa Sensibilitätseinschränkungen machen.

Alle Patient:innen sollten während der Untersuchung zum 3. Untersuchungsgang wach und orientiert sein. Sollte dies in den ersten 6–12 h nach dem Unfall nicht gegeben sein, so müssen der 3. Untersuchungsgang trotzdem durchgeführt sowie objektivierbare Befunde erhoben und dokumentiert werden. Er sollte dann allerdings wiederholt werden, sobald der Patient/die Patientin relevant wacher und/oder orientierter ist. Beatmete Patient:innen sollten bei der Untersuchung so wach wie möglich sein. *Konsequenterweise erfolgt nach der Extubation immer ein weiterer 3. Untersuchungsgang.*

Einen Sonderfall stellen Patient:innen mit Wirbelsäulenverletzungen dar. Die Information, ob und wie ausgeprägt eine Schädigung des Rückenmarks vorliegt, ist von entscheidender Bedeutung für das weitere therapeutische und operative Vorgehen der nächsten Stunden [10]. Dies gilt sowohl bei bisher unversorgten Wirbelsäulenverletzungen als auch direkt nach operativer Versorgung. Deshalb sollte bei beatmeten, sedierten Patient:innen mit einer Verletzung der Wirbelsäule so früh wie möglich eine klinische Beurteilung der peripheren Neurologie, idealerweise unter Reduktion der Sedierung, durchgeführt werden.

Zur Dokumentation von Rückenmarkverletzungen sollten standardisierte Untersuchungsbogen verwendet werden, z. B. das ASIA ISNCSCI-Protokoll [1].

### Inhalt des 3. Untersuchungsganges

Die gründliche Untersuchung im Rahmen des 3. Untersuchungsganges zielt darauf ab, ein vollständiges Bild sämtlicher Verletzungsfolgen zu erhalten. Hierzu sollen bereits bekannte Befunde gewürdigt und eine auftretende Dynamik dokumentiert werden. Neu aufgetretene oder bislang nicht erfasste Befunde sollen ebenfalls dokumentiert werden. Die Untersuchung erfolgt strukturiert, z. B. von Kopf nach Fuß. Eigen- und Fremdanamnese sowie Hilfsmittel wie z. B. Ultraschall kommen ergänzend hinzu. Die Ergebnisse des 3. Untersuchungsganges sollen dokumentiert werden, und wenn sich aus diesen diagnostische oder therapeutische Konsequenzen ergeben, so sollen diese eingeleitet werden.

### Zusätzliche Schritte über die Anamnese und Untersuchung hinaus

#### „Clear the pelvis“

Bei Patient:innen mit einer vermuteten Verletzung des Beckenrings sollte ein Beckengurt bereits prähospital angelegt werden [2]. Im Regelfall werden der Primary Survey und die sich anschließende CT-Untersuchung mit liegendem Beckengurt erfolgen [9].

Eine mechanisch instabile Beckenverletzung, welche zu einer hämodynamischen Instabilität führt, sollte im Rahmen des Primary Survey detektiert werden und führt in der Regel zu einer unmittelbaren Intervention im Sinne einer operativen oder interventionellen Blutstillung, meist in Kombination mit einer mechanischen Stabilisierung des Beckenrings durch Beckenzwinge oder supraacetabulären Fixateur externe [5].

Wenn in der Traumaspirale eine knöcherne Verletzung des Beckens, die zu einer Instabilität des Beckenrings führt, nachgewiesen wird, sollte der Beckengurt bis zur operativen Versorgung belassen werden.

Zeigt sich in der Traumaspirale keine knöcherne Verletzung des Beckenrings, so sollte der Beckengurt unter anliegendem Monitoring geöffnet und eine Untersuchung des Beckens, ggf. auch eine zusätzliche konventionell radiologische Bildgebung, zum sicheren Ausschluss einer ligamentären Open-Book-Verletzung erfolgen [9]. Im Tertiary Survey muss die vorhandene Diagnostik bezüglich einer möglicherweise vorliegenden Verletzung des Beckenrings evaluiert werden.

#### Komplettierung aller relevanten Informationen

Zusätzlich zu Anamnese, körperlicher Untersuchung und ergänzender apparativer Diagnostik sollten für die weitere Behandlung relevante Informationen jetzt endgültig zusammengetragen werden. Handelt es sich um einen Arbeitsunfall/ein Gewaltverbrechen, muss gegebenenfalls die Polizei eingeschaltet werden, müssen Angehörige informiert oder ermittelt werden? Ist eine Schwangerschaft sicher ausgeschlossen, der Tetanusschutz sicher vorhanden oder eine Auffrischung im Schockraum erfolgt? Kann bereits jetzt ein Screening auf psychische Belastung erfolgen und/oder psychologische Mitbeurteilung oder Hilfe eingeleitet werden?

#### Einordnung der Befunde

Die erhobenen Befunde müssen mit den bereits vorhandenen Befunden abgeglichen werden. Insbesondere muss unverzüglich in Zusammenschau mit der vorhandenen Bildgebung entschieden werden, ob ergänzende Untersuchungen erforderlich sind. Die im Schockraum-CT erhobenen Befunde werden vice versa mit den klinischen Befunden abgeglichen, ggf. mit den in der Zwischenzeit erhobenen Befunden aus Eigen- und Fremdanamnese und Vorbefunden der Hausärztin/des Hausarztes. Die Einordnung der Befunde sollte durch einen Arzt/eine Ärztin mit Facharztstandard in einem für die Traumaversorgung relevanten Fach erfolgen.

#### Dokumentation

Zur Dokumentation kann ein speziell hierfür konzipierter Bogen genutzt werden. Bei sehr ausführlichen Befunden muss zusätzlich auf die üblichen Dokumentationsprozesse der Klinik zurückgegriffen werden. Wünschenswert wäre eine vollständige Integration des Bogens in das Krankenhausinformationssystem (KIS) bzw. PDMS der Station.

Haut- und Weichteilverletzungen sollten großzügig mittels Fotos und ggf. sogar Video dokumentiert werden. Hierbei ist auch zu bedenken, dass die Dokumentation häufig später bei der juristischen Aufarbeitung des Unfalls oder der Ursache der Verletzungen genutzt wird [7].

Wie andere medizinische Befunde unterliegt eine solche Dokumentation der ärztlichen Schweigepflicht. Eine unbefugte Weitergabe an die Ermittlungsbehörden oder an Dritte ist daher nicht zulässig [7].

#### Festlegung des weiteren Prozedere

Der (ggf. mehrfach durchgeführte) 3. Untersuchungsgang ermöglicht, neu auftretende Befunde sowie die Dynamik vorhandener Verletzungsfolgen kontinuierlich zu bewerten und somit das für den individuellen Patienten/die individuelle Patientin festgelegte Vorgehen regelmäßig zu reevaluieren und entweder zu bestätigen oder zu modifizieren: Müssen weitere Befunde erhoben werden, Bildgebung, Kompartmentdruckmessung, konsiliarische Mitbeurteilungen durch andere Fachabteilungen? Muss ein engeres Überwachungsintervall festgelegt werden, z. B. 4‑stündliche Messungen des intraabdominellen Drucks? Muss ein bereits erstelltes Konzept der sekundären OP reevaluiert werden, z. B. Änderung der Dringlichkeiten bei verschiedenen Extremitätenverletzungen aufgrund von Weichteil- oder Gefäß-Nerven-Schäden?

## Aktueller Stand in Deutschland

Den Autor:innen ist keine Erhebung zu Praxis und Durchdringung des Tertiary Surveys in Deutschland bekannt. Im Rahmen der Zertifizierung von Traumazentren werden keine Überprüfungen des diesbezüglichen Prozesses durchgeführt. Aus der Sicht der Autor:innen ist eine klinikinterne Verfahrensanweisung zum Tertiary Survey dringend erforderlich.

### Schritte zur Implementierung eines standardisierten Tertiary Survey

Für die Durchführung eines Tertiary Surveys empfiehlt sich die Verwendung eines Untersuchungsbogens, der sowohl als Checkliste für den/die Untersucher/in als auch zur Dokumentation dient. Eine digitale Lösung ist anzustreben, wobei die Möglichkeit zur bettseitigen Nutzung wichtig ist. Eine Einbindung in das jeweilige KIS bzw. PDMS ist zu fordern. Ebenso ist der Tertiary Survey als Teil der digitalen Notfallkette im Rahmen der digitalen Dokumentation von Notfällen vom Unfallort bis zur Rehabilitation einzubinden.

Die genaue Ausgestaltung eines Bogens, unabhängig davon, ob digital oder analog, kann grundsätzlich individuell erfolgen und sich den lokalen bereits bestehenden Gegebenheiten anpassen. Das betrifft die Detailliertheit der Checkliste bezüglich einzelner zu untersuchender Parameter und auch die genaue Methode, z. B. mit welchem Messinstrument Wachheit und Sedierungstiefe oder Delir gemessen werden. Ein standardisierter Untersuchungsbogen kann als Vorlage dienen und bietet den Vorteil der Vergleichbarkeit.

Die Frage, welche/r Arzt/Ärztin aus welcher Fachabteilung den vollständigen Tertiary Survey durchführt, muss den individuellen Strukturen des Traumazentrums angepasst werden. Die Bewertung der erhobenen Befunde mit der Festlegung der daraus resultierenden Konsequenzen sollte durch einen Arzt/eine Ärztin mit Facharztstandard in einem für die Traumaversorgung relevanten Fach erfolgen.

### Entwicklung eines standardisierten Untersuchungsbogens durch die Sektion Trauma der DIVI

Die Sektion Trauma der DIVI hat es sich zur Aufgabe gemacht, einen Vorschlag für einen konkreten Untersuchungsbogen zu entwerfen und kontinuierlich weiterzuentwickeln (Tab. 1).

**Tab. 1:** Inhalte des Tertiary Surveys, A: klinische Untersuchung, B: weitere Aufgaben, Administration, Dokumentation

Bereich	Inhalte/Körperregionen	Methoden/Hinweise
*A: Klinische Untersuchung*		
Allgemeinzustand	Wachheit, Orientierung, Sedierungstiefe, Agitation	Klinische Beurteilung, Scores
Körperliche Untersuchung	Kopf, Hals, Thorax, Abdomen, Rücken, Wirbelsäule, Becken, obere und untere Extremitäten	Inspektion, Palpation, ggf. Auskultation, Perkussion, Sonographie
Gefäßstatus	Periphere Gefäße	Palpation, ggf. Sonographie
Neurologische Untersuchung	Hirnnerven, Rückenmark, periphere Motorik und Sensibilität	Klinische Prüfung, ggf. ASIA-Protokoll
Clear the Pelvis	Entscheidung über weiteres Vorgehen bei Beckenverletzung	Strategie bei temporärer Stabilisierung
*B: Weitere Aufgaben und Dokumentation*		
Evaluation vorhandener (radiologischer) Befunde	Vollständigkeit der Bildgebung, ggf. Ergänzungen	–
Reevaluation weiterer Befunde	Tetanusschutz, Schwangerschaftstest	–
Anamnese komplettieren	Allergien, Vorerkrankungen, Vormedikation	Eigen- und Fremdanamnese
Administrative Daten	Kontaktdaten, Angehörige, Hausarzt, Hinweise auf Straftat? Arbeitsunfall? Eilbetreuung, Fotodokumentation	Ggf. Information von Angehörigen, BG, Polizei; ggf. Einleitung einer Betreuung
Einschätzung der seelischen Belastung	Suizidalität, psychische Belastung	Ggf. Hinzuziehen von Psychiatrie/Psychosomatik, Psychologie
Prozedere festlegen	Weitere Diagnostik, Untersuchungsintervalle, therapeutisches Vorgehen	Multidisziplinäre Abstimmung

**Abb. 1:**
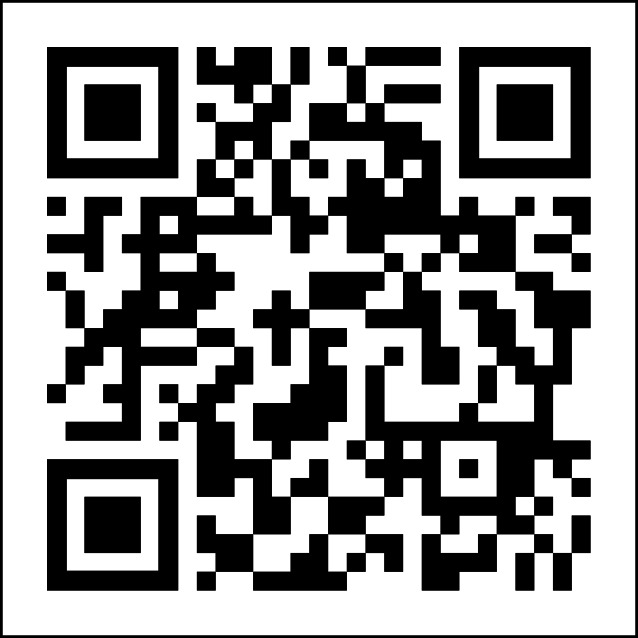
In der Sektion konsentierter Untersuchungsbogen Bogen auf der DIVI-Homepage zur freien Nutzung; abrufbar über diesen QR-Code und unter https://www.divi.de/sektionen/trauma

Hierzu wurde ein in der Sektion konsentierter Bogen auf der DIVI-Homepage zur freien Nutzung veröffentlicht (verfügbar als Zusatzmaterial online oder über Abb. 1).

Nutzende sind aufgefordert, aktiv an der kontinuierlichen Verbesserung des Bogens durch Feedback mitzuwirken. In regelmäßigen Abständen wird das Feedback durch die Sektion evaluiert und der Bogen im Konsens angepasst.

Langfristiges Ziel ist die wissenschaftlich begleitete Optimierung eines notwendigen Prozesses bei der Versorgung polytraumatisierter Patienten. Wie bei kontinuierlichen Verbesserungen des Notfallprotokolls der DIVI sind die Ziele eine bestmögliche Versorgung der Patienten durch größtmögliche Reduktion von Missed Injuries, medizinisch sinnvolle, anwenderfreundliche Dokumentation, Implementierung des Tertiary Survey in PDMS sowie Implementierung in Qualitätsmanagement, inklusive Nutzbarkeit für Register und Forschung.

## Fazit für die Praxis

- Der Tertiary Survey sollte bei allen Traumapatientinnen und -patienten, die einen Primary und Secondary Survey nach ATLS-Standard erhalten haben, durchgeführt werden.
- Die erstmalige Durchführung des Tertiary Survey sollte 6–12 h nach dem Traumaeintritt erfolgen, je nach Befund sollte er (ggf. auch mehrfach) wiederholt werden.
- Es sollte eine standardisierte Dokumentation (z. B. über den DIVI-Bogen), wenn möglich mit Integration ins KIS erfolgen.
- Ziele sind die strukturierte Dokumentation sämtlicher Verletzungsfolgen und weiterer relevanter Informationen, das frühzeitige Erkennen initial übersehener Verletzungen sowie neu aufgetretener oder progredienter Befunde sowie die Reevaluation des Behandlungsplanes.

## Supplementary Information

Untersuchungsbogen für den Tertiary Survey zum Ausdrucken. Mit freundlicher Genehmigung der DIVI.
