# Supplementary material for: Tertiary survey for trauma patients: Practical recommendations of the Trauma Section of the DIVI
Source: Unfallchirurgie (Heidelb). 2025 Sep 1;128(10):801–5. [Article in German] doi: 10.1007/s00113-025-01600-y (PMC12454486; doi:10.1007/s00113-025-01600-y)
Supplement: Supplementary file 1 — Untersuchungsbogen für den Tertiary Survey zum Ausdrucken. Mit freundlicher Genehmigung der DIVI. [file 113_2025_1600_MOESM1_ESM.pdf]

Untersuchungsbogen
Tertiary Survey
zum Ausdrucken

DIVI
Deutsche Interdisziplinäre Vereinigung
für Intensiv- und Notfallmedizin

Patientenetikett

**Untersuchungsdatum**	
**Uhrzeit der Untersuchung**	
**Unfalldatum**	
**BG/SAV-Fall eingeleitet?**	

Relevante Vorbefunde aus Primary und Secondary Survey/erfolgte Bildgebung:

Untersuchung

**GCS**	**E:**	**V:**	**M:**

	opB	auffällig	ggf. Beschreibung	neu	vorbekannt	Bildgebung nötig?
**Kopf**						
Prellmarken/Wunden						
Ohren						
Augen						
Visus						
Pupillen						
Zähne						
Nase						
Kehlkopf						
**Wirbelsäule**						
Prellmarken/Wunden						
Querschnitt			Falls ja → **ASIA-Protokoll**			
**Thorax**						
Prellmarken/Wunden						
Hautemphysem						
Auskultation						
**Abdomen**						
Prellmarken/Wunden						
Auskultation						
FAST-Sonogaphie						
Peritonismus						
Hämaturie						
**Rücken**						
Prellmarken/Wunden						
Urogenital						
Rektale Untersuchung						
Hämaturie						
**Becken**	**Clear the pelvis:** Ligamentäre Verletzung ausgeschlossen? →					
Prellmarken/Wunden						
(Druck)Schmerz						

		opB	auffällig	ggf. Beschreibung	neu	vorbekannt	Bildgebung nötig?
**Extremitäten (oben)**							
rechts	Schulter rechts						
	Oberarm rechts						
	Ellenbogen rechts						
	Unterarm rechts						
	Handgelenk rechts						
	Hand rechts						
	Sensibilität OE rechts						
	Motorik OE rechts						
	A.radialis rechts						
	A. ulnaris rechts						
links	Schulter links						
	Oberarm links						
	Ellenbogen links						
	Unterarm links						
	Handgelenk links						
	Hand links						
	Sensibilität OE links						
	Motorik OE links						
	A.radialis links						
	A. ulnaris links						
**Extremitäten (unten)**							
rechts	Oberschenkel rechts						
	Kniegelenk rechts						
	Unterschenkel rechts						
	OSG rechts						
	Fuß rechts						
	Sensibilität UE rechts						
	Motorik UE rechts						
	A. tib. post. rechts						
	A. dors. ped. rechts						
links	Oberschenkel links						
	Kniegelenk links						
	Unterschenkel links						
	OSG links						
	Fuß links						
	Sensibilität UE links						
	A. tib. post. links						
	A. tib. post. links						
	A. dors. ped. links						

Seelische Belastung?	
Suizidalität?	

Psychologie-Konsil angemeldet?	
Psychiatrie-Konsil angemeldet?	

Sonstiges	ja	nein	Anmerkungen
Angehörige informiert			
Vorerkrankungen erfragt			
Tetanus			
Antibiose			
Schwangerschaft			
Fotodoku erfolgt			
Vorsorgevollmacht?			
Eilbetreuung notwendig?			

Kontakt Angehörige

Kontakt Hausarzt

Weiteres Prozedere/Ausstehende Diagnostik:
